# Real-World Comparison of Maribavir to Foscarnet for the Treatment of Cytomegalovirus in Solid Organ and Hematopoietic Stem Cell Transplant Recipients

**DOI:** 10.3390/v16121889

**Published:** 2024-12-07

**Authors:** Lauren Ogawa, Chelsea Morinishi, Ashrit Multani, Pryce Gaynor, Omer E. Beaird, Christine Pham, Joanna M. Schaenman

**Affiliations:** 1 Division of Infectious Diseases, Department of Medicine, David Geffen School of Medicine at UCLA, Los Angeles, CA 90095, USA; lyanagimoto-ogawa@mednet.ucla.edu (L.O.); cmorinishi@mednet.ucla.edu (C.M.);; 2 Department of Pharmacy, David Geffen School of Medicine at UCLA, Los Angeles, CA 90095, USA

**Keywords:** CMV, resistance, maribavir, foscarnet, immunocompromised

## Abstract

Cytomegalovirus (CMV) infection in solid organ transplant (SOT) and hematopoietic cell transplant (HCT) recipients may increase the risk of rejection or allograft dysfunction, other infection(s), and morbidity and mortality. Treatment can be challenging due to medication-associated toxicities. Maribavir (MBV) is a promising option for the treatment of resistant or refractory (R/R) CMV infection in lieu of foscarnet (FOS), which has long been the recommended therapy for (val)ganciclovir-resistant infection. This was a single-center retrospective study of clinical outcomes of patients who received MBV compared to a control group who received FOS for an episode of CMV infection. Each cohort consisted of 27 episodes of CMV infection. Twenty patients in the MBV cohort and from the FOS cohort cleared the infection, with five and three patients developing MBV or FOS resistance, respectively. There were no statistically significant differences in failure of therapy as evidenced by persistent DNAemia (*p* = 0.56) or development of antiviral resistance (*p* = 0.24). In conclusion, MBV was as effective as FOS for the treatment of R/R CMV infection and was better tolerated without increased risk of antiviral resistance.

## 1. Introduction

Despite currently available antiviral therapy, cytomegalovirus (CMV) remains a challenging opportunistic infection to treat, with significant morbidity and mortality in solid organ transplant (SOT) and hematopoietic cell transplant (HCT) recipients. First line antiviral agents, valganciclovir (VGC) and ganciclovir (GCV), are myelotoxic [1]. Other antiviral options, such as foscarnet (FOS) and cidofovir (CDV), have multiple toxicities, including nephrotoxicity, and typically require administration with close monitoring in an inpatient setting [2]. Even with effective dosing for prophylaxis and treatment, prolonged exposure to antiviral medications and incomplete suppression of CMV may contribute to the development of antiviral resistance mutations [3].

Refractory or resistant (R/R) CMV infection, which can occur independently or concurrently, confers worse clinical outcomes, from increased rates of rejection, allograft failure, hospitalizations, and mortality [4,5,6,7]. Resistant CMV infection refers to the detection of genetic mutation (s) that predict decreased susceptibility to antiviral therapy, whereas refractory CMV infection is defined by the persistence of symptoms or increased viral load by one log10 after two weeks of appropriately dosed therapy [8,9]. Risk factors for R/R CMV infection include T-cell depletion, lack of CMV-specific immunity, intense immunosuppressive therapy, poor absorption of antiviral therapy, sub-optimal antiviral dosing, prolonged antiviral exposure, prior antiviral exposure, high CMV viral load, and intermittent low-level CMV DNAemia [8].

Maribavir (MBV) is a novel agent with demonstrated efficacy in achieving clearance of DNAemia in R/R CMV infection without the myelotoxicity associated with VGC or GCV or the nephrotoxicity associated with FOS or CDV [10,11]. Prior to the introduction of MBV, FOS had been the recommended therapy for resistant CMV or for those who may not tolerate GCV or VGC [1,5]. A phase III clinical trial demonstrated superior efficacy of MBV achieving viral clearance in R/R CMV infection compared with investigator-assigned therapy after 8 weeks of treatment in SOT and HCT recipients and was maintained for 4 weeks post-completion of therapy [12]. However, there are less data on the real-world efficacy of MBV, especially in high-level CMV DNAemia. A few small studies have reviewed the utility of MBV, but there have been no direct comparisons with other antiviral agents for R/R CMV infection [13,14].

To address this question, we evaluated the clinical outcomes of SOT and HCT recipients at a high-volume transplant center to determine the effectiveness and safety of MBV compared to a control group who received FOS for CMV infection.

## 2. Materials and Methods

This was a single-center retrospective cohort study of SOT and HCT recipients who received MBV for the treatment of CMV infection at the University of California, Los Angeles (UCLA) Health Centers. To identify an appropriate comparison control group, we queried our electronic medical record system for patients who received FOS for an episode of CMV infection. The study was approved by the Institutional Review Board.

**Figure 1:**
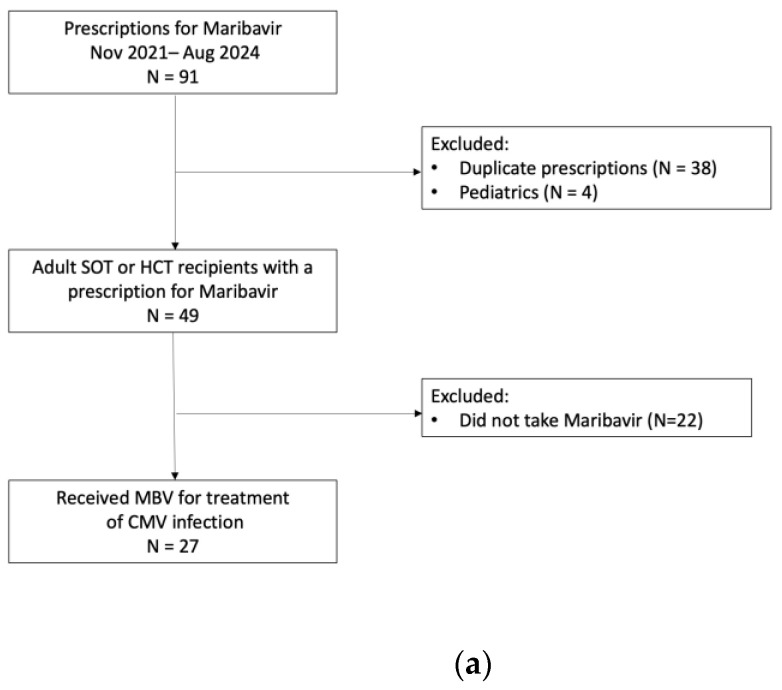

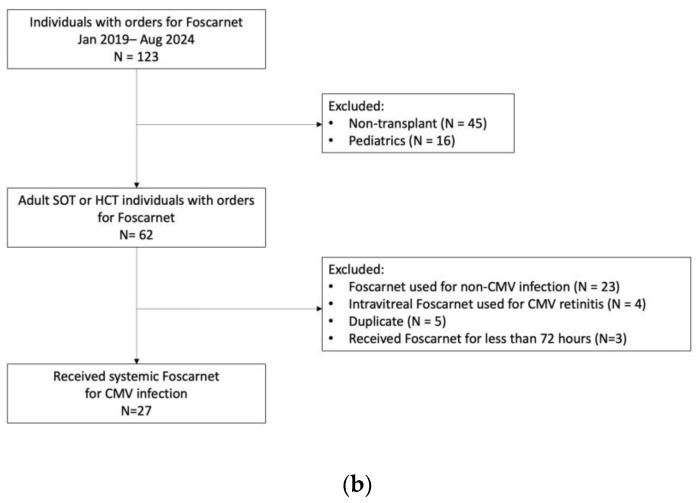
(**a**). Flow diagram of exclusion criteria for the maribavir cohort. (**b**). Flow diagram of exclusion criteria for foscarnet cohort.

Two internal databases of individuals with prescriptions or orders for MBV from 1 November 2021 to 31 August 2024 and FOS from 1 January 2019 to 31 August 2024 were reviewed. In both groups, pediatric patients (under age 18), duplicate orders or prescriptions, and those who received therapy for less than 72 h were excluded. From the MBV group, patients who were prescribed MBV but did not actually take MBV were excluded. From the FOS group, non-transplant recipients, individuals who received FOS for a non-CMV infection, and individuals who did not receive systemic FOS were excluded (Figure 1a,b).

Electronic medical records were reviewed, and data collected included demographic information, type of transplant, immunosuppression (IS), antiviral prophylaxis, peak CMV viral load, CMV T-cell immunity panels, CMV genotypic resistance testing, antiviral treatment, antiviral adverse effects, co-infection(s), and mortality. Bloodwork focusing on absolute neutrophil count (ANC), absolute lymphocyte count (ALC), renal function, and electrolytes (potassium, calcium, magnesium, and phosphorous) was also compiled from time of initiation, switch, and completion of CMV therapy. Renal dysfunction was defined as a 25% or greater change in glomerular filtration rate. The collected characteristics and outcomes were based on episode of CMV infection, with the exception of mortality. For individuals with multiple episodes of CMV infection, only the final episode of infection was counted in the mortality analysis.

Typical post-transplant prophylaxis regimens have been described elsewhere, and all SOT recipients received CMV prophylaxis corresponding to the organ(s) transplanted and donor/recipient (D/R) serostatus risk based on American Society of Transplantation (AST) and international guidelines [1,15,16]. For SOT recipients, risk was defined as high risk (D+/R-), intermediate risk (R+), and low risk (D-/R-). For HCT recipients, high risk was defined as R+. Duration of prophylaxis varied with the type of transplanted organ from 3 months to life-long based on risk. All CMV seropositive HCT recipients received CMV prophylaxis until day +100 in accordance with internal protocols. Any adjustment in prophylaxis and use of alternative agents were made at the discretion of an Infectious Diseases physician or primary transplant physician. MBV prescriptions were for 400 mg orally twice daily. FOS was dosed at 90 mg/kg intravenously every 12 h and adjusted for renal function by an Infectious Diseases physician.

Asymptomatic CMV DNAemia was defined as the detection of CMV DNA in plasma using quantitative polymerase chain reaction (PCR) alone [1,17]. CMV disease was defined as either proven, probable, or possible end-organ disease based on the 2024 consensus definitions of CMV infection and disease in transplant patients [17]. Successful treatment response was defined as the resolution of any presenting symptoms and clearance of DNAemia with one quantitative plasma CMV PCR test that was either negative or detectable but below the level of quantification. Relapse or recurrent DNAemia or disease was defined as a positive plasma CMV PCR with or without symptoms occurring within 8 weeks of stopping MBV or FOS. Resistant CMV was defined as the detection of gene mutations resulting in diminished susceptibility to an antiviral [8].

All CMV quantitative PCR testing was performed at the UCLA Clinical Microbiology Laboratory. Testing was performed using Roche AmpliPrep CMV PCR assay. In October 2023, CMV quantitative PCR testing switched to Roche Cobas 6800/880 CMV PCR assay with higher analytical sensitivity and the level of quantification changed from <137 IU/mL to <35 IU/mL. CMV drug resistance testing was performed at reference laboratories, ARUP or Eurofins Viracor. CMV T-cell immunity assays were performed via intracellular cytokine staining by flow cytometry (Viracor Eurofins CMV inSIGHT™ T Cell Immunity Testing). Statistical analysis was performed on JMP Pro-17. Numeric variables were analyzed using a nonparametric approach. Categorical variables were analyzed by an unpaired two-sample Wilcoxon test. Statistical significance was defined as *p* < 0.05. Given the small size of the cohort studied, a multivariate analysis was not performed, as it could lead to overfitting of the statistical models.

## 3. Results

### 3.1. Demographic and Clinical Characteristics

Twenty-seven SOT and HCT recipients with asymptomatic CMV DNAemia or disease received MBV and were included in the final analysis (Figure 1a). Baseline demographics and characteristics were similar between the two groups (Table 1). There were no statistically significant differences between the two groups regarding age, sex, race, type of transplant, or IS. The majority of patients were on triple IS consisting of tacrolimus, mycophenolate, and prednisone.

There were 27 unique patients in the MBV group. In the FOS group, we identified 27 episodes of FOS administration for the treatment of asymptomatic CMV DNAemia or disease (Figure 1b). Two patients had more than one episode of CMV infection that was treated with FOS. In the MBV cohort, 18/27 (63%) were on CMV prophylaxis at time of infection. The most commonly used prophylaxis was VGC (12, 66.7%), followed by letermovir (LET, 4, 22.2%) and acyclovir (ACV, 2, 11.1%). In the FOS cohort, the majority of patients (23/27, 85.2%) were on CMV prophylaxis at the time of infection. Similar to the MBV group, the most commonly used agent was VGC (16/27, 69.6%). LET (3/27, 13%) and ACV (1/27, 4.3%) were also used as prophylaxis. Three patients (13%) were on maintenance MBV at time of CMV infection.

### 3.2. Initial CMV Management and Assessment of Resistance

In both the MBV and FOS cohorts, the majority of patients had asymptomatic CMV DNAemia and were initially treated with GCV or VGC (Table 2). Five patients received MBV as the initial treatment due to pre-existing leukopenia or history of intolerance to first-line therapy. In contrast, 10 patients received FOS as the initial treatment. The reasons for choosing FOS as the initial therapy over VGC or GCV were due to concerns for resistant CMV, given evidence of breakthrough on prophylaxis or pre-existing cytopenia.

The median time to switch from initial therapy to MBV was 22 days. At the time of switch in therapy, median ANC was 1.9 × 10^3^ cells/mL and ALC was 0.84 × 10^3^ cells/mL. In general, patients demonstrated a trend towards receiving first-line therapy for a longer period of time before switching to FOS, with a median duration of 38 days, although this difference did not reach statistical significance (*p* = 0.158). At the time of switch in therapy, median ANC was 1.9 × 10^3^ cells/mL and ALC was 0.68 × 10^3^ cells/mL. T-cell immunity panels were not uniformly collected in either group but were more commonly ordered in the FOS cohort (52%) than the MBV cohort (19%) (*p* = 0.002). Of those who did have a CMV T-cell immunity panel, the majority had an immune response below 0.2%, with no significant difference in terms of CD4 or CD8 response between the MBV versus FOS patients (*p* = 0.77 and *p* = 0.91, respectively). Resistance testing was performed in 93% (25/27) of cases from the MBV cohort (Table 3). Of those, 17 (68%) were found to have resistance, most commonly at UL97 conferring resistance to VGC/GCV. Two had resistance to LET with UL97 C325W. In the FOS cohort, resistance testing was performed in all patients, which revealed UL97 or UL54 resistance genes in 24 patients (85.2%) conferring VGC/GCV and in some cases CDV and MBV resistance. In both groups, moderate to high VGC/GCV UL97 resistance at codons 460, 520, 594, 595, and 603 was detected.

### 3.3. Virologic and Clinical Response to MBV and FOS

Twenty patients in the MBV cohort had resolution of infection, with two experiencing recurrence as measured by CMV DNAemia within 8 weeks of stopping therapy (Table 2). Seven patients were continued on a longer course of MBV as either suppression or prophylaxis, with two developing breakthrough CMV DNAemia. Median duration to clearance was 23 days, but patients remained on therapy for a median time of 44 days.

Patients received FOS for a median of 21 days, with 66.7% (18/27) clearing infection in a median time of 16 days (Table 2). Five had recurrence of DNAemia within 8 weeks of stopping therapy. The time of FOS administration was shorter than the time receiving MBV (*p* = 0.01), which was largely related to issues regarding the need for intravenous administration and concern for toxicity. There was no significant difference in failure defined as persistent DNAemia by viral load at the time of the switch in patients receiving either MBV (*p* = 0.68) or FOS (*p* = 0.27).

In the comparison analysis, there was no statistically significant difference in drug failure as evidenced by persistent DNAemia (*p* = 0.56). In the MBV cohort, eight patients had repeat resistance testing (one due to the development of breakthrough infection while on secondary prophylaxis and seven due to the inability to clear DNAemia, Table 3). Five were found to have MBV resistance and were switched to FOS; of these five patients, four demonstrated virologic clearance and one died. In the FOS cohort, six patients had repeat resistance testing (three due to refractory asymptomatic CMV DNAemia and three due to refractory end-organ disease). Of the six patients, three had developed resistance to FOS (two with end-organ disease and one with asymptomatic CMV DNAemia). MBV resistance occurred at UL97 T409M, H411Y, and C480F. FOS resistance was identified at UL54 A809V, G841A, and Q578H.

There was no difference in the risk of developing resistance to therapy while receiving MBV or FOS (*p* = 0.64). These cases of resistance to FOS occurred prior to the introduction of MBV in the market, so one case was enrolled in a clinical trial, one was switched to CDV+GCV, and one had no change in therapy due to mortality. Three patients were switched from FOS to MBV in order to avoid intravenous therapy or potential side effects.

### 3.4. Adverse Effects

In terms of adverse effects, seven patients (25.9%) reported dysgeusia with MBV. Eight patients on FOS reported adverse effects (8/27, 29.6%), which included nausea (four), headaches (two), and genital ulcers (two). Strikingly, twenty-three FOS patients, (85.2%) compared with zero MBV patients, experienced either electrolyte imbalances or renal dysfunction (*p* < 0.001). Two FOS patients required a switch or hold in therapy due to renal dysfunction.

### 3.5. Mortality

All-cause mortality occurred in six patients (22.2%) from the MBV cohort. One fatality was suspected to be due to complications of post-transplant lymphoproliferative disease and R/R CMV disease with colitis and gastrointestinal bleeding.

All-cause mortality was observed after eight episodes of CMV (29.6%) from the FOS group; in four of those, complications of CMV disease were thought to be a contributing factor. Complications were mostly related to end-organ disease, such as gastrointestinal bleeding with CMV colitis or respiratory failure with CMV pneumonia and co-infection. There was no significant difference in all-cause mortality between groups (*p* = 0.14).

## 4. Discussion

The 2021 FDA approval of MBV added another therapeutic option for the treatment of CMV. MBV has the potential advantages of not causing myelotoxicity or nephrotoxicity, is available as an oral formulation, and does not require inpatient administration or frequent lab monitoring. Despite multiple treatment options, CMV infection remains a common and sometimes challenging complication amongst SOT and HCT recipients. A criticism of the SOLSTICE study was the concern for external validity with using MBV for the treatment of R/R CMV [18]. Using a real-world cohort, we compared and evaluated treatment outcomes for CMV infection using MBV or FOS.

To the best of our knowledge, this is the largest real-world cohort of MBV in comparison to conventional therapy with FOS. In this study, MBV appeared to be as effective but better tolerated than FOS. Although we were not able to show superiority as seen in the SOLSTICE study, MBV remains a viable treatment option in this real-world analysis and there were no statistically significant differences in rates of treatment failure [12]. Unlike the SOLSTICE study, where subjects were treated with MBV for 8 weeks, it is common practice at our institution to treat CMV infection until resolution of clinical symptoms and clearance of DNAemia with one negative (or below the level of detection) plasma CMV PCR. We found that patients were treated for a median time of 44 days with MBV and had a median time to clearance of 20 days. There was also a lower rate of relapse in the MBV group compared to the FOS group within 8 weeks of stopping therapy. This may be due to the receipt of extended MBV therapy in patients deemed to be at risk for relapse with shorter courses of therapy. Alternatively, this observation may be explained by our center’s standard practice of reducing IS during episodes of CMV infection, while immunosuppression modifications were not clearly described in the SOLSTICE study.

Our study cohort is also larger than other real-world non-comparison studies that have reviewed outcomes with MBV [13,14,19]. These studies have all raised concerns about virologic failure during treatment with MBV and the development of MBV-specific resistance. In this study, although there was a trend towards higher rates of MBV resistance (18.5%), this was not a statistically significant difference when compared to rates of FOS resistance (11.1%). Another issue raised by the SOLSTICE study was that the study population primarily consisted of patients with low CMV viral loads, which has raised the question of the efficacy of MBV in those with high levels. We found no negative impact in terms of treatment success or failure by viral load in patients receiving MBV. However, it is possible that clinicians avoided MBV use in patients with higher viral load, given the concern that high viral load correlates with immune incompetence. Future directions would include additional immunologic testing to determine whether immune incompetence was a primary reason behind failure.

Ongoing comparison studies can help shed light on preferred agents for prophylaxis and treatment of asymptomatic disease, end-organ disease, or R/R CMV infection. A low ANC and/or ALC may reflect either CMV disease or a negative impact of GCV/VGC, which may in turn further negatively impact the immune control of CMV. Therefore, it is possible that an earlier switch to a non-myelotoxic regimen would be beneficial, but this is an area that needs further study.

Our study was limited due to the small cohort size, heterogeneous patient population, and retrospective design. Although there were no significant baseline differences between groups, a larger sample size would have enabled a multivariable analysis to control for possible confounding factors, such as peak viral load, CMV end-organ disease, or asymptomatic DNAemia. We were also limited to the interpretation of clinical documentation regarding indications for choice of antivirals, reasons for switches in therapy, and use of oral versus intravenous formulations. In addition, our study population was based on individuals who had prescriptions at our institution and may have missed patients who received a prescription for MBV from an outside pharmacy. The optimal duration of antiviral treatment was individualized based on clinician discretion. Some patients were continued on secondary prophylaxis with MBV and subsequently developed breakthrough infection. More data is necessary to guide the optimal dosing and duration of treatment with MBV while limiting the risk of developing resistance mutations. Future studies are also needed to evaluate the impact of transplant type, high viral loads, and CMV end-organ disease on MBV treatment outcomes. However, these limitations are somewhat mitigated, as this was a single-center study with access to granular data regarding CMV infection, laboratory abnormalities, and response to therapy. Furthermore, the use of standardized internal protocols across time for all transplant recipients led to our ability to identify MBV and FOS cohorts that were well-matched in terms of demographic and clinical characteristics.

In summary, in a real-world cohort, MBV was used to successfully treat CMV infection with an increased success rate at our center compared with published data. Compared to FOS, MBV demonstrated no significant difference in the development of resistance and also had a trend towards a lower rate of recurrent infection within 8 weeks of completing therapy. MBV therefore presents an attractive alternative antiviral option for the treatment of R/R CMV infection and/or for patients who are unable to tolerate conventional therapy since it appears to be as effective and better tolerated than FOS.

## Figures and Tables

**Table 1:** Characteristics and demographics of maribavir and foscarnet cohorts.

Characteristic		Maribavir (*n* = 27)	Foscarnet (*n* = 27)	*p*-Value
Age, median (range)		59 (29–75)	60 (18–74)	0.80
Sex, *n* (%)	Male	13 (48.15)	16 (59.3)	0.59
	Female	14 (51.85)	11 (40.7)	
Race, *n* (%)	Asian	3 (11.11)	3 (11.1)	0.65
	Black or African-American	2 (7.41)	4 (14.8)	
	White	10 (37.04)	12 (44.4)	
	Hispanic or Latino	12 (44.44)	8 (29.6)	
Type of Transplant, *n* (%)	SOT	19 (70.37)	22 (81.5)	0.53
	HCT	8 (29.63)	5 (18.5)	
	Heart	1 (3.70)	3 (11.1)	
	Lung	9 (33.33)	13 (48.1)	
	Liver	2 (7.41)	0 (0)	
	Kidney	7 (25.93)	6 (22.2)	
	alloHCT	7 (25.93)	5 (18.5)	
	autoHCT	1 (3.70)	0 (0)	
Treated for rejection, GVHD, or relapsed disease, *n* (%)		3 (11.11)	2 (7.40)	0.39
Immunosuppression, *n* (%)	Four or more	1 (3.70)	0 (0)	0.95
	Triple therapy	19 (70.37)	22 (81.5)	
	Dual therapy	4 (14.81)	5 (18.5)	
	One agent	2 (7.41)	0 (0)	
CMV Serostatus Risk, *n* (%)	High or Moderate	25 (92.59)	22 (81.5)	0.22
	Low	2 (7.41)	5 (18.5)	
On CMV prophylaxis at time of episode of infection, *n* (%)		18 (62.96)	23 (85.2)	0.11
First occurrence of CMV infection, *n* (%)		16 (59.26)	11 (33.3)	0.06
Recurrent CMV infection, *n* (%)		11 (40.74)	16 (66.7)	0.17

Abbreviations: CMV, cytomegalovirus; HCT, hematopoietic cell transplant; SOT, solid organ transplant.

**Table 2:** Presentation of CMV infection and treatment outcomes.

Presentation and Outcomes		Maribavir (*n* = 27)	Foscarnet (*n* = 27)	*p*-Value
Peak viral load, median (range)		10,538 (444–250,709)	24,184 (1100–889,657)	0.49
Asymptomatic CMV DNAemia		24 (88.9)	21 (81.5)	0.27
Initial Treatment, *n* (%)	Valganciclovir	17 (63.0)	12 (44.4)	<0.0001
	Ganciclovir	5 (18.5)	5 (18.5)	
	Maribavir	5(18.5)	--	
	Foscarnet	--	10 (37)	
Number of failed regimens before switch to MBV or FOS, *n* (%)	Started with MBV or FOS	5 (18.5)	10 (37)	0.52
	Failed 1	13 (48.2)	8 (29.6)	
	Failed 2	9 (33.3)	7 (25.9)	
	Failed 3	0 (0)	2 (7.4)	
Treatment duration with MBV or FOS, median days (range)		44 (11–126)	21 (4–148)	0.01
Time until clearance of DNAemia, median days (range)		23 (2–67)	16 (4–65)	0.65
DNAemia cleared, *n* (%)		20 (74.1)	18 (66.7)	0.55
Recurrence within 8 weeks of stopping MBV (*n* = 20) or FOS (*n* = 18), *n* (%)		2 (10.0)	5 (38.9)	0.13
Developed resistance, *n* (%)		5 (18.5)	3 (11.1)	0.64

Abbreviations: CMV, cytomegalovirus; FOS, foscarnet; MBV, maribavir.

**Table 3:** Distribution of antiviral drug-resistance mutations arising during maribavir or foscarnet treatment.

Antiviral Treatment	Gene	Amino Acid Substitution	Predicted Antiviral Resistance	*n*
MBV	UL97	T409M	MBV	3
MBV	UL97	H411Y	MBV	2
MBV	UL97	C480F	MBV, GCV	1
FOS	UL54	G841A	FOS, CDV, GCV	1
FOS	UL54	Q578H	FOS, CDV, GCV	1
FOS	UL54	A809V	FOS	1

## Data Availability

The data that support the findings of this study are available on request from the corresponding author. The data are not publicly available due to privacy and ethical restrictions and the local institution’s data policy.

## References

[1] Kotton C.N., Kumar D., Caliendo A.M., Huprikar S., Chou S., Danziger-Isakov L., Humar A. (2018). The Transplantation Society International CMV Consensus Group The Third International Consensus Guidelines on the Management of Cytomegalovirus in Solid-organ Transplantation. Transplantation.

[2] Fishman J.A. (2017). Infection in Organ Transplantation. Am. J. Transplant..

[3] Young P., Rubin J., Angarone M., Flaherty J., Penugonda S., Stosor V., Ison M. (2016). Ganciclovir-resistant cytomegalovirus infection in solid organ transplant recipients: A single-center retrospective cohort study. Transpl. Infect. Dis..

[4] Liu J., Kong J., Chang Y., Chen H., Chen Y., Han W., Wang Y., Yan C., Wang J., Wang F. (2015). Patients with refractory cytomegalovirus (CMV) infection following allogeneic haematopoietic stem cell transplantation are at high risk for CMV disease and non-relapse mortality. Clin. Microbiol. Infect..

[5] Yong M.K., Shigle T.L., Kim Y.-J., Carpenter P.A., Chemaly R.F., Papanicolaou G.A. (2021). American Society for Transplantation and Cellular Therapy Series: #4—Cytomegalovirus treatment and management of resistant or refractory infections after hematopoietic cell transplantation. Transplant. Cell. Ther..

[6] Karantoni E., Zavras P.D., Su Y., Fang J., Tamari R., Cho C., Perales M.-A., Stern A., Papanicolaou G.A. (2022). Outcomes of refractory Cytomegalovirus infection in the first year after allogeneic hematopoietic cell transplantation. Transplant. Cell. Ther..

[7] Heliövaara E., Husain S., Martinu T., Singer L.G., Cypel M., Humar A., Keshavjee S., Tikkanen J. (2019). Drug-resistant cytomegalovirus infection after lung transplantation: Incidence, characteristics, and clinical outcomes. J. Heart Lung Transplant..

[8] Chemaly R.F., Chou S., Einsele H., Griffiths P., Avery R., Razonable R.R., Mullane K.M., Kotton C., Lundgren J., Komatsu T.E. (2019). Definitions of resistant and refractory Cytomegalovirus infection and disease in transplant recipients for use in clinical trials. Clin. Infect. Dis..

[9] Kotton C.N., Kamar N. (2023). New insights on CMV management in solid organ transplant patients: Prevention, treatment, and management of resistant/refractory disease. Infect. Dis. Ther..

[10] Walti C.S., Khanna N., Avery R.K., Helanterä I. (2023). New treatment options for refractory/resistant CMV infection. Transpl. Int..

[11] Papanicolaou G.A., Silveira F.P., Langston A.A., Pereira M.R., Avery R.K., Uknis M., Wijatyk A., Wu J., Boeckh M., Marty F.M. (2019). Maribavir for refractory or resistant Cytomegalovirus infections in hematopoietic-cell or solid-organ transplant recipients: A randomized, dose-ranging, double-blind, phase 2 study. Clin. Infect. Dis..

[12] Avery R.K., Alain S., Alexander B.D., Blumberg E.A., Chemaly R.F., Cordonnier C., Duarte R.F., Florescu D.F., Kamar N., Kumar D. (2022). Maribavir for Refractory Cytomegalovirus Infections With or Without Resistance Post-Transplant: Results From a Phase 3 Randomized Clinical Trial. Clin. Infect. Dis..

[13] Ni B., Wolfe C.R., Arif S., Carugati M., Heldman M.R., Messina J.A., Miller R.A., Saullo J.L., Baker A.W., Maziarz E.K. (2024). Real-world experience with maribavir for treatment of Cytomegalovirus infection in high-risk solid organ transplant recipients. Open Forum Infect. Dis..

[14] Fung M., DeVoe C., Spottiswoode N., Doernberg S.B. (2023). Maribavir for Cytomegalovirus treatment in the real world-not a silver bullet. Open Forum Infect. Dis..

[15] Razonable R.R., Humar A. (2019). Cytomegalovirus in solid organ transplant recipients-Guidelines of the American Society of Transplantation Infectious Diseases Community of Practice. Clin. Transplant..

[16] Ogawa L., Multani A., Beaird O.E., Gaynor P., Carlson M., Garner O.B., Schiller G., Schaenman J.M. (2024). Risk factors and outcomes of Mucorales infection in a modern cohort of solid organ transplant, hematopoietic cell transplant, and chimeric antigen receptor T-cell therapy recipients. Transplant. Proc..

[17] Ljungman P., Chemaly R.F., Khawaya F., Alain S., Avery R., Badshah C., Boeckh M., Fournier M., Hodowanec A., Komatsu T. (2024). Consensus Definitions of Cytomegalovirus (CMV) Infection and Disease in Transplant Patients Including Resistant and Refractory CMV for Use in Clinical Trials: 2024 Update From the Transplant Associated Virus Infections Forum. Clin. Infect. Dis..

[18] Canadian Agency for Drugs and Technologies in Health (2023). Maribavir (Livtencity): CADTH Reimbursement Review: Therapeutic Area: Post-Transplant Cytomegalovirus Infection.

[19] Corcione S., Lupia T., Vita D., Sidoti F., Zanotto E., Solidoro P., Biancone L., Costa C., Balagna R., De Rosa F.G. (2024). Maribavir treatment for resistant cytomegalovirus disseminated disease in kidney transplant recipients: A case-based scoping review of real life data in literature. Transplant. Rev..

